# Effect of pre-use of Dexmedetomidine on the effective inhibitory dose of remimazolam tosilate on positive cardiovascular response in double-lumen endobronchial intubation: a clinical study

**DOI:** 10.1186/s12871-023-02305-8

**Published:** 2023-11-23

**Authors:** Shiyou Wei, Xiaojuan Liu, Rui Chang, Xue Chen, Tao Zheng, Jingyun Wang, Huqing Liu, Faqiang Zhang, Jiong Song, Xin Lv

**Affiliations:** 1 Department of Anesthesiology, Shanghai Pulmonary Hospital, School of Medicine, Tongji University, 507 Zhengmin Rd, Shanghai, 200433 China; 2 Medical department, Shanghai Pulmonary Hospital, School of Medicine, Tongji University, 507 Zhengmin Rd, Shanghai, 200433 China

**Keywords:** Remimazolam Tosilate, Dexmedetomidine, Double-lumen Endobronchial Intubation, Cardiovascular Response, ED50/ED95

## Abstract

**Background:**

Herein, the effect of pre-use of Dexmedetomidine(Dex) on the half-effective dose (ED50) and 95%-effective dose (ED95) of Remimazolam tosilate(RT) in inhibiting the positive cardiovascular response(CR) which means blood pressure or heart rate rises above a critical threshold induced by double-lumen bronchial intubation was evaluated.

**Methods:**

Patients who underwent video-assisted thoracic surgery were divided into groups A (0), B (0.5 µg/kg), and C (1 µg/kg) based on different Dex doses. Group A included subgroups comprising young (A-Y) and elderly (A-O) patients. Neither groups B nor C included elderly patients due of the sedative effect of Dex. Based on the previous subject’s CR, the dose of RT was increased or decreased in the next patient using the sequential method. This trial would be terminated when the seventh crossover occurred, at which point the sample size met the stable estimate of the target dose. Heart rate (HR) and mean arterial pressure (MAP) were monitored throughout the trial, and sedation was assessed using the Modified Observer’s Assessment of Alertness/Sedation (MOAA/S) scale. HR and MAP were recorded at baseline (T1), the end of Dex (T2), and the end of RT (T3), the maximum HR and MAP were recorded within 3 min of intubation from beginning to end (T4). There was a positive CR when the T4 levels rose above 15% of the baseline. The ED50/ED95 and corresponding confidence interval were calculated using probability regression.

**Results:**

In total, 114 patients completed the trial. Without the use of Dex, the ED50/ED95 of TR inhibiting the positive CR caused by double-lumen bronchial intubation was 0.198/0.227 and 0.155/0.181 mg/kg in groups A-Y and A-O, respectively. The changes in vital signs from T1 to T3 were similar in the subgroups, indicating that the elderly patients were more sensitive to the dose of RT. The ED50/ED95 of RT inhibiting the positive CR caused by double-lumen endobronchial intubation was 0.122/0.150 and 0.068/0.084 mg/kg in groups B and C, respectively. And, the fluctuation of blood pressure from T3 to T4 was reduced by using Dex. RT was 100% effective in sedation with no significant inhibition of circulation. Apart from one case of hypotension occurred in group A-Y, two cases of low HR in group B, and one case of low HR in group C, no other adverse events were noted.

**Conclusions:**

The optimal dose of RT to inhibit positive CR induced by double-lumen bronchial intubation in elderly patients was 0.18 mg/kg and 0.23 mg/kg in younger patients. When the pre-use dose of Dex was 0.5 µg/kg, the optimal dose to inhibit positive CR of RT was 0.15 mg/kg. And, when the pre-use dose of Dex was 1 µg/kg, the optimal dose of RT was 0.9 mg/kg.

**Clinical trial registration:**

NCT05631028.

**Supplementary Information:**

The online version contains supplementary material available at 10.1186/s12871-023-02305-8.

## Introduction

To ensure the smooth progression of thoracic surgery, double-lumen endobronchial intubation is the main method of pulmonary isolation technology and also an important anesthetic method [1]. This method involves rotating the catheter within the trachea and inserting it into one side of the main bronchus across the protuberance, which is deeper and thicker than single-lumen endotracheal intubation. Meanwhile, the bronchial catheter needs to be accurately positioned, which often makes intubation and operation much more difficult and time-consuming, leading to greater stimulation in patients and more CRs [2]. When blood pressure or heart rate rises above a critical threshold that may create a risk, it is called a positive cardiovascular response. This critical value is often defined as 15% above the base level [3, 4]. Positive CR, due to its dramatic blood pressure and heart rate changes, will increase the risk of circulatory complications, such as myocardial ischemia, arrhythmia, and even heart failure, and result in a poor prognosis in the perioperative period [5].

An ideal anesthesia induction procedure includes no intraoperative consciousness, effective analgesia, muscle relaxation, no stress response, and slight disruption to the circulation. However, there is no present drug that can exert all of the above-mentioned effects. Therefore, a combination of drugs is commonly used for anesthesia induction. Common regimens include a combination of intravenous anesthetics, opioids, muscle relaxants, and adjuvants. The primary component of several anesthesia regimens is intravenous anesthesia. Propofol, the most commonly used intravenous anesthetic, has the advantages of rapid onset, rapid awakening, and complete recovery. However, respiratory depression and decreased blood pressure [6] are the most significant adverse reactions along with injection pain, myoclonus, and injection-site phlebitis. Another short-acting intravenous anesthetic etomidate has little effect on hemodynamics and mild respiratory depression. However, pain at the injection site can be as high as 10–50%, and muscle tremors, muscular rigidity, and similar convulsions can be noted during anesthesia induction in severe cases. Furthermore, long-term use can impair adrenal cortex function [7]. Thus, anesthetics with a rapid onset of action, good control of anesthesia depth, and adequate, rapid, and predictable awakening, particularly with a small hemodynamic impact, and one that does not rely on hepatic and renal metabolism have become the most desired drugs for anesthesia induction and maintenance.

A novel ultrashort-acting benzodiazepine RT is metabolized to an inactive metabolite through plasma nonspecific esterases [6] and can be safely used for general anesthesia induction and maintenance, with little effect on respiration and circulation and no injection pain. It is an ideal anesthetic alternative as its metabolism does not pass through the liver or kidney with no damage to liver and kidney function [8, 9]. Due to the short application of this new drug in general anesthesia and the lack of clinical data in all aspects, it is important to clinically clarify its reasonable use in the anesthesia induction process. The currently most commonly used adjuvant drug in clinical anesthesia Dex is a highly selective α2 adrenergic receptor agonist, its effects include sedation, hypnosis, anti-anxiety, stress reduction, hemodynamic stabilization, analgesia, salivary gland secretion inhibition, chill resistance, as well as a reduction in postoperative cognitive dysfunction complications [10]. However, there is sparse clinical data on Dex combined with RT for anesthesia induction.

Therefore, the aim of this study was to determine the effect of the pre-use of Dex on the ED50and ED95 of RT in inhibiting the positive CR induced by double-lumen bronchial intubation, hoping to provide more data for the effective dose of RT and provide references for rational clinical use.

## Methods

### Study Design

This was a prospective, single-center, sequential-design clinical trial using Dixon up-and-down methods. The first subject was administered a dose close to ED95, and the CR of the first subject was used to determine the dose for the second subject. When a positive cardiovascular response occurs, the fixed dose will be increased for the next patient. When the cardiovascular response is negative, the next patient dose decreases the fixed dose. This design does not have an accurate sample calculation method, but 20 to 40 sample sizes can form a stable estimate of the target dose [11–13]. In the up-and-down process, Dixon [14] suggested that the test could be terminated after a fixed number of crossovers (i.e., crossover between positive and negative reactions) and the results met the need for stable estimation [12]. Researchers generally believe that 6 crossovers are sufficient [12, 15]. Therefore, this trial will be terminated when the seventh crossover occurred. This study was approved by the Clinical Research Center of Shanghai Pulmonary Hospital (ID: 2022LY0416) and the Ethics Review Committee of Shanghai Pulmonary Hospital (ID: L22-261) and was registered on 30/11/2022(NCT05631028) in clinicaltrial.gov before patient recruitment. It was conducted in accordance with the principles of the *Declaration of Helsinki.* All patients provided written informed consent.

### Patients

Patients who underwent partial pneumonectomy via thoracoscopy at Shanghai Pulmonary Hospital during December 2022 were included. The inclusion criteria were as follows: (1) Patients who underwent optional video-assisted thoracoscopic surgery (VATS), (2) those aged more than 18 years old, and (3) Those with an American Association of Anesthesiologists grade of I–III. The exclusion criteria were as follows: (1) Patients with a systolic pressure of ≥ 160 mmHg or a diastolic pressure of ≥ 110 mmHg or an HR of ≥ 110 beats/min in the operating room at rest, (2) those with long-term preoperative use of analgesia or sedation drugs, (3) those with pregnancy, lactation, pregnancy possibility, and planned pregnancy, (4) those with an allergy history of the test drug, and (5) those with mental illness or an inability to communicate normally.

### Arms

The patients were divided into three groups based on the doses of pre-used Dex. The dose of Dex in group A was zero, suggesting that Dex was not used. The patients in group A were divided into the young (A-Y) group (aged 18–64 years) and the elderly (A-O) group (aged ≥ 65 years) by age. Group B received 0.5 µg/kg Dex over a period of 10 min, and group C received 1 µg/kg Dex over a period of 10 min.

To achieve the same anesthetic effect, older patients often require smaller doses. It is necessary for us to define the effective dose of drugs in the elderly population, so the population is divided into elderly and young people at the age of 65 years [16, 17]. Elderly patients often experience drowsiness and even loss of consciousness with group B or C doses of dexmedetomidine. So both groups B and C did not include elderly patients.

### Anesthesia procedure and interventions

The blood pressure, ECG, and pulse oxygen saturation were routinely monitored by Philips Patient Monitor (IntelliVue MX800), venous access was established, and invasive monitoring was performed as needed after entering the operating room. Non-invasive cuff blood pressure was recorded for analysis in this study. The process was initiated with 10 min infusion of Dex [18](Hengrui Co., Jiangsu, China).

After the Dex infusion was completed, anesthesia induction was performed. Sufentanil(Hengrui Co., Jiangsu, China) 0.5 µg/kg was intravenously administered, followed by RT(Hengrui Co., Jiangsu, China) 2 min later. The starting dose of RT was 0.25 mg/kg in group A-Y, 0.15 mg/kg in group A-O, 0.15 mg/kg in group B, and 0.1 mg/kg in group C according to the pretest results. The dynamic adjustment unit was 0.01 mg/kg in each group. The starting dose was different for each group. To achieve the same anesthetic effect, older patients often require smaller doses. And, there is a synergy between the intravenous anesthetic drugs. The higher the dose of Dex, the smaller the required dose of RT. For different populations and scenarios of each group, the starting dose was different. For the interpretation of the pretest, an additional file shows this in more detail [see *Additional file 1*].

Next, 0.6 mg/kg Rocuronium(Xianju Co., Zhejiang, China) was administered following the loss of consciousness (MOAA/S ≤ 1). When the muscle relaxant was completely effective, double-lumen endotracheal intubation(Covidien Co.,Minneapolis,USA) was performed under direct vision, the catheter position was determined using fiberoptic bronchoscopy. Either a prolonged intubation process or an intubation failure will affect the results. Tracheal intubation was performed by an experienced anesthesiologist, with the aid of visualization. If the intubation process for more than 2 min or the intubation failed, the test will be terminated immediately.

Propofol(Fresenius Kabi Co., Beijing, China) was pumped at 4–8 mg/kg/h, Remifentanil(Hengrui Co., Jiangsu, China) was pumped at 0.05–0.1 µg/kg/min, and Rocuronium was intermittently added for anesthesia maintenance. On the ventilation side, a protective ventilation strategy with a low tidal volume (4–6 mL/kg) was used along with positive end-expiratory pressure ventilation and a pulmonary resuscitation strategy. The intraoperative infusion used sodium acetate Ringer’s solution and hydroxyethyl starch with the ratio was 2:1, and the speed was 5–8 mL/kg/h.

Ondansetron(Qilu Co., Shandong, China) 4 mg and Flurbiprofen axetil(Taide Co., Beijing, China) 50 mg were scheduled to be administered 20 min preoperatively. Following anesthesia, the patient-controlled analgesia pump was connected to the vein, and a volume of 2 mL of 1 µg/mL Sufentanil with a background dose of 2 µg/h was given. Patient-controlled analgesia with a locking time of 15 min and 0.5 mL per patient-controlled analgesia administration. Paracetamol tablets (Qilu Co., Shandong, China) 0.5 g were administered orally for remedial analgesia.

### Outcome measures

The primary outcome of this study was ED50 and ED95 of RT to inhibit the positive CR which was induced by double-lumen bronchial intubation during anesthesia induction after Dex be pre-used. The inhibition of positive CR by RT means that patients will not experience positive CR induced by intubation stimulation after the use of RT.

The secondary outcome was a change in CR with the evaluation time included the start of endotracheal intubation until three minutes after completion. A positive CR was defined as follows: MAP or elevation in the HR of ≥ 15% of the baseline or tachycardia (> 120 beats/min) or hypertension (systolic blood pressure > 180 mmHg) during intubation. Non-invasive blood pressure (systolic, diastolic, and MAP) and HR were measured at the following four time points: the baseline time (T1) when patients entered the operating room at rest; (T2) at the end of Dex pumping; (T3) at the end of RT injection, and (T4) 3 min within tracheal intubation, wherein MAP = diastolic + 1/3 (systolic-diastolic) and MAP and HR were calculated and compared. Sedation success was defined as success when MOAA/S ≤ 1, indicating that the patient was losing consciousness. Hypotension was defined as a systolic blood pressure < 90 mmHg or a MAP < 60 mmHg. Low HR was defined as a heart rate < 50 beats per minute.

### Statistical analysis

Normally distributed data were expressed as mean ± standard deviation, non-normally distributed data were expressed as median (interquartile range) and counting data as number (percentage). SPSS 25.0 software(IBM Co., New York, USA ) was used for statistical analysis, and the Kolmogorov–Smirnov test was used to test the normality of the data. The measurement data were compared in pairs using analysis of variance (least significant difference) or a nonparametric method. The counting data were tested using the χ^2^ test based on whether the data met the normality and homogeneity of variance requirements.

Using the Dixon up-and-down method, terminate the trial when the seventh crossover occurred. Probit regression was used to fit the trial results, with drug dose as the dependent variable and the occurrence of positive results as the outcome. Fit the equation that is closest to the actual situation and use it to estimate the ED50, ED95, and corresponding 95% confidence interval(CI) for each group. This has also been used in previous studies [12, 19]. P < 0.05 was considered statistically significant.

## Results

**Fig. 1:**
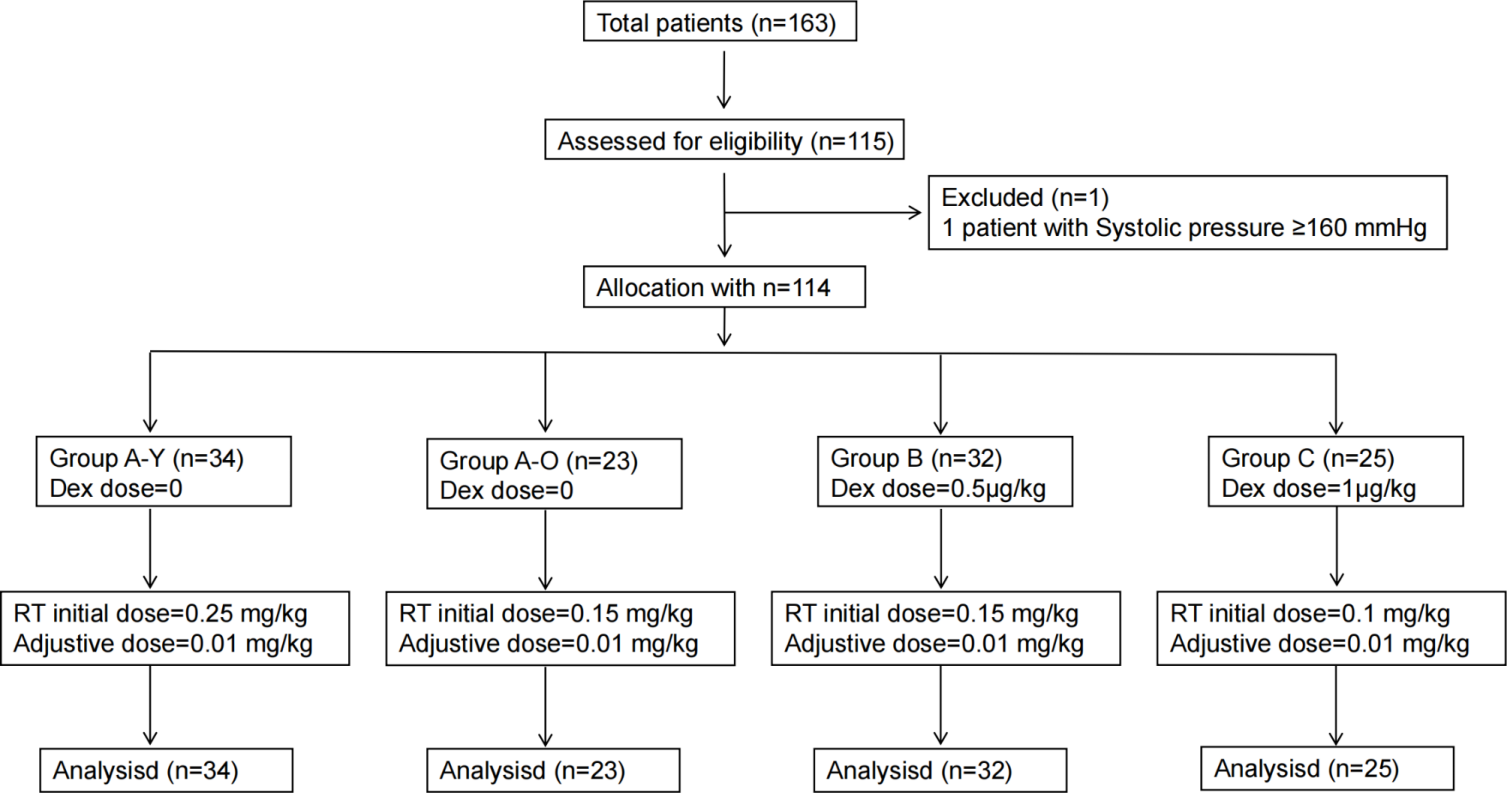
Patient flowchart. Dex: dexmedetomidine, RT: Remimazolam tosilate

A total of 163 patients underwent preoperative evaluation and 48 patients were not enrolled in this study cohort. In total, 115 patients were included, with one patient being admitted to the operating room with a systolic blood pressure still greater than 160 mmHg at rest and thus being excluded. Table 1 shows the patients’ vital signs. See Fig. 1 for the flowchart.

**Table 1:** Characteristics of the patients

Variables	Group A - Y (n = 34 )	Group A - O (n = 23 )	Group B (n = 32 )	Group C (n = 25 )
Age (y, Mean ± SD)	50.1 ± 13.3	70.0 ± 3.0	53.0 ± 8.7	53.5 ± 40.4
Gender(%male)	12(35.3)	14(60.9)	11(34.4)	12(48)
Weight (kg, Mean ± SD)	63.2 ± 10.1	66.3 ± 11.5	62.8 ± 9.1	66.6 ± 10.0
Height (m, Mean ± SD)	1.6 ± 0.1	1.6 ± 0.1	1.6 ± 0.1	1.7 ± 0.1
BMI (kg/m^2^, Mean ± SD)	23.9 ± 3.2	24.6 ± 3.3	23.6 ± 2.6	24.1 ± 2.6
ASA grade (1/2/3)	(24/10/)	(7/14/2)	(22/10/)	(17/8/)

ASA: American Society of Anesthesiologists

### ED50 and ED95 of inhibitory effects of RT on positive CR in double-lumen endobronchial intubation

**Fig. 2:**
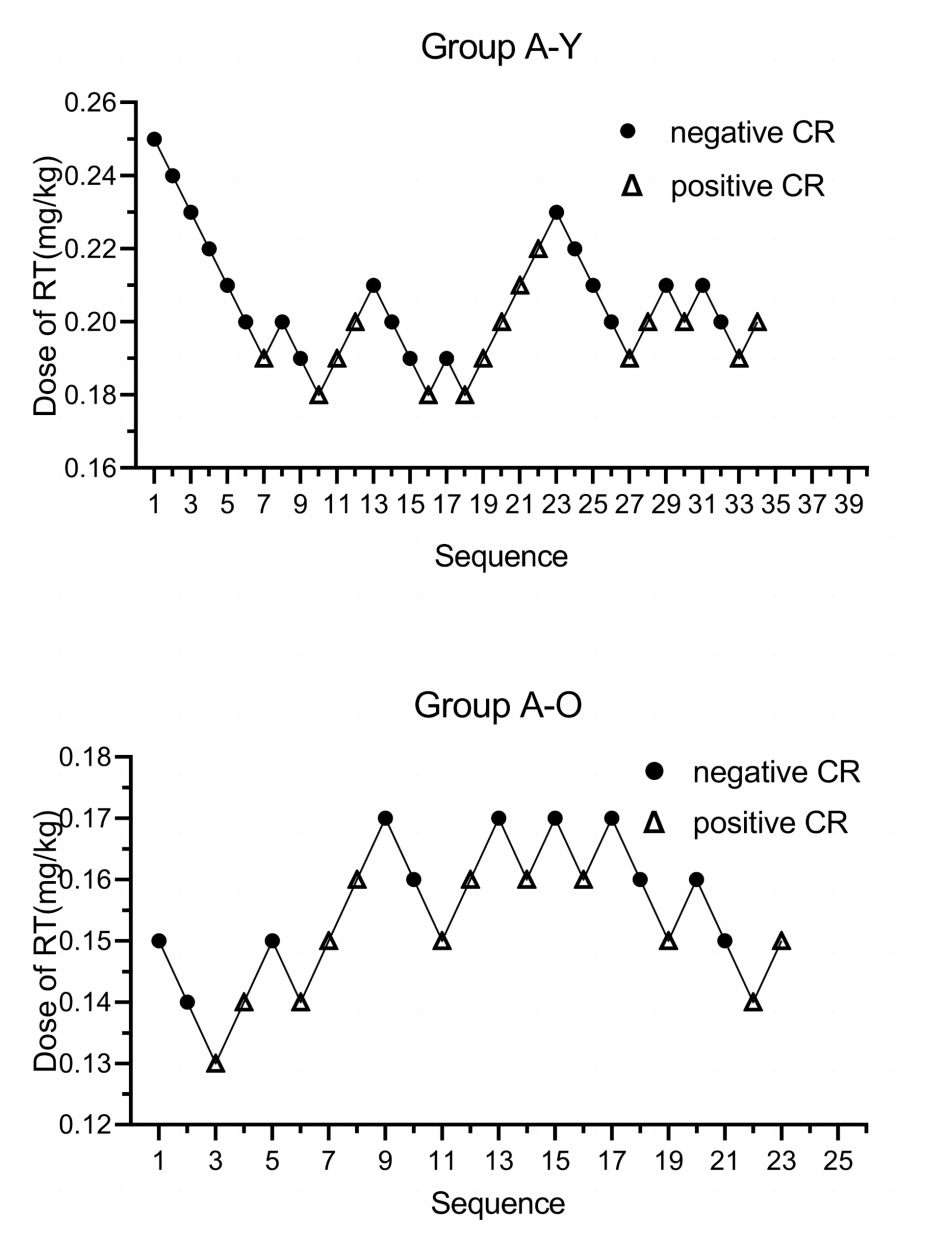
Sequential plots of group A, the ED50 of RT inhibiting the positive CR in double-lumen bronchial intubation in group A-Y was 0.198 mg/kg (95% CI: 0.181–0.208) and the ED95 was 0.227 mg/kg (95% CI: 0.214–0.308). The ED50 was 0.155 mg/kg (95% CI: 0.135–0.185) and the ED95 was 0.181 mg/kg (95% CI: 0.167–0.548) in group A-O. RT: Remimazolam tosilate, CR:Cardiovascular Response

There were 57 patients in group A, including 34 patients in group A-Y, with 19 patients with a negative CR and 15 with a positive CR, 23 patients in group A-O, with 11 patients with a negative CR and 12 with a positive CR. The ED50 of RT for inhibition on positive CR in double-lumen endobronchial intubation in group A-Y was 0.198 mg/kg (95% CI: 0.181–0.208) and the ED95 was 0.227 mg/kg (95% CI: 0.214–0.308). The ED50 was 0.155 mg/kg (95% CI: 0.135–0.185) and ED95 was 0.181 mg/kg (95% CI: 0.167–0.548) in the A-O group (Fig. 2).

**Fig. 3:**
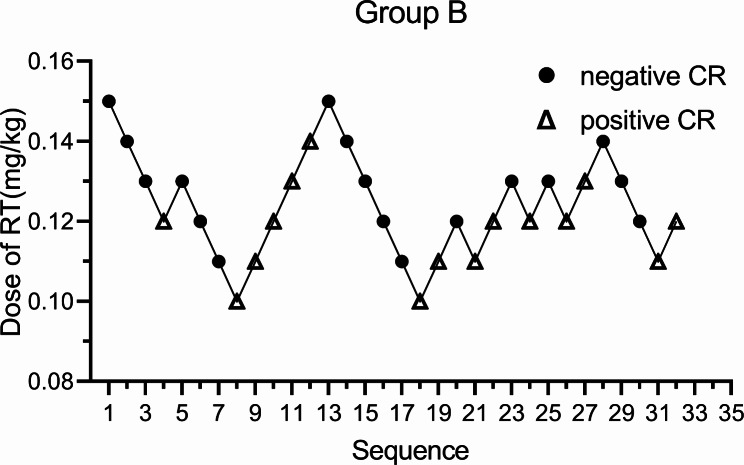
Sequential plots of group B, the ED50 of RT inhibiting the positive CR in double-lumen endobronchial intubation was 0.122 mg/kg (95% CI: 0.108–0.133) and the ED95 was 0.15 mg/kg (95% CI: 0.137–0.239). RT: Remimazolam tosilate, CR:Cardiovascular Response

There were 32 patients in group B, 17 with a negative CR and 15 with a positive CR. The ED50 of group B was 0.122 mg/kg (95% CI: 0.108–0.133) and the ED95 was 0.15 mg/kg (95% CI: 0.137–0.239) (Fig. 3).

**Fig. 4:**
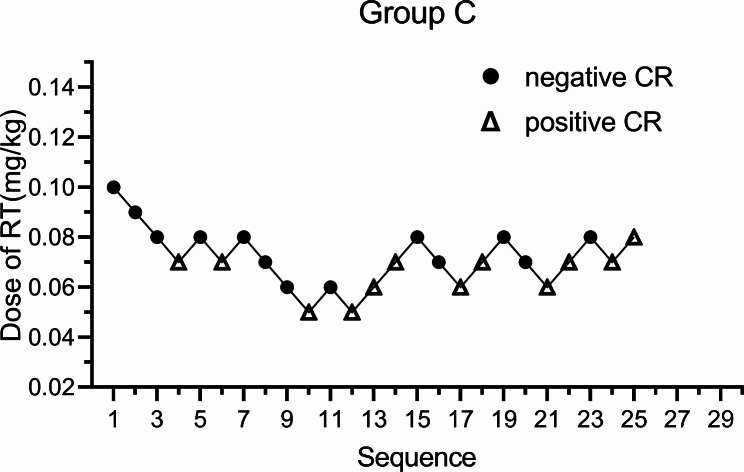
Sequential plots of group C, the ED50 of RT inhibiting the positive CR in double-lumen bronchial intubation was 0.068 mg/kg (95% CI: 0.059–0.076) and the ED95 was 0.084 mg/kg (95% CI: 0.076–0.130). RT: Remimazolam tosilate, CR:Cardiovascular Response

There were 25 patients in group C, 13 with a negative CR and 12 with a positive CR. The ED50 of group C was 0.068 mg/kg (95% CI: 0.059–0.076) and ED95 was 0.084 mg/kg (95% CI: 0.076–0.130) (Fig. 4).

### Blood pressure and HR fluctuations during anesthesia induction

The MAP and HR of each group at four time points are shown in Table 2. The analysis of variance and inter-group comparison of MAP and HR in groups A, B, and C at T1 revealed P > 0.05, suggesting that there was no statistical difference in the vital signs at baseline.

**Table 2:** MAP and HR at four time points

	Time Point	Group A	Group A-O	Group A-Y	Group B	Group C
MAP(mmHg)	T1	96.4 ± 8.3^a^	99.0 ± 8.2	94.7 ± 8.1	95.2 ± 7.0^a^	99.4 ± 6.4^a^
	T2	/	/	/	88.1 ± 9.0	94.8 ± 9.7
	T3	82.2 ± 8.2	83.7 ± 8.8	81.1 ± 7.8	83.1 ± 10.3	91.1 ± 10.5
	T4	101.7 ± 13.9	105.4 ± 13.2	99.3 ± 14.1	99.7 ± 14.9	99.6 ± 10.3
h(bpm)	T1	79.1 ± 10.6^a^	78.8 ± 8.7	79.3 ± 11.8	77.1 ± 8.4^a^	76.0 ± 8.7^a^
	T2	/	/	/	66.3 ± 8.7	62.8 ± 6.7
	T3	76.3 ± 11.0	76.6 ± 9.3	76.0 ± 12.2	66.8 ± 9.6	64.0 ± 7.6
	T4	90.8 ± 12.2	92.6 ± 12.1	89.5 ± 12.3	81.2 ± 10.4	80.0 ± 10.9

MAP: mean arterial pressure, HR: heart rate; ^a^ P>0.05 vs. T1.

When groups A-Y and A-O were compared, differences in mean arterial pressure (dMAP) (13.6 ± 9.2 vs. 15.3 ± 8.8, P = 0.485) and differences in heart rate (dHR) (3.3 ± 11.3 vs. 2.2 ± 7.3, P = 0.697) revealed no significant difference in the decline of vital signs from T1 to T3. Since group A-O received a lower dose of RT than group A-Y, it is possible that rational drug use could reduce the circulatory inhibition of the patients in group A-O.

Group A-Y versus group B, dMAP (18.2 ± 13.5 vs. 16.7 ± 10.1, P = 0.614) and dHR (13.5 ± 11.8 vs. 14.4 ± 10.9, P = 0.739); group A-Y versus group C, dMAP (18.2 ± 13.5 vs. 8.5 ± 11.2, P = 0.005) and dHR (13.5 ± 11.8 vs. 16.1 ± 11.2, P = 0.4). Dex was not used in group A-Y, and the increase in blood pressure before and after intubation was greater than that in groups B and C. The P value was < 0.05 (dMAP) in group A-Y versus group C, suggesting that Dex reduced the CR in intubation and stabilized the circulation.

### Successful sedation and incidence of hypotension and low HR

All patients had MOAA/S ≤ 1, and the sedation was 100% effective. After the pre-use of Dex, only one case of hypotension occurred in group A-Y, two cases of low HR in group B, and one case of low HR in group C (Table 3). No hypotension and bradycardia occurred during the time period for assessing CR.

**Table 3:** Successful sedation and the incidence of hypotension and low heart rate

	Group A - Y (n = 34 )	Group A - O (n = 23 )	Group B (n = 32 )	Group C (n = 25 )
Success sedation (%)	34(100)	23(100)	32(100)	25(100)
Hypotension (%)	1(2.94)	0	0	0
Low heart rate (%)	0	0	2(6.25)	1(4)

## Discussion

In thoracic surgery, general anesthesia via double-lumen endobronchial intubation is a primary anesthesia technique. The large size of the double-lumen catheter along with the rotation and position of the catheter in the trachea during intubation often lead to an intense cardiovascular response(CR).

As a new intravenous anesthetic, RT has many advantages, such as no damage to liver and kidney function and minimal impact on hemodynamics.The present study investigated the effective dose of RT combined with Dex on the inhibition of positive CR induced by double-lumen endobronchial intubation. The dose of RT varies for patients of various ages, and the pre-use of Dex reduces the dose of RT in inhibiting the positive CR, filling the gap in RT research.

The most commonly used anesthetic adjuvant that can stabilize circulation and reduce CR is Dex [20]. Furthermore, it was found to significantly reduce the dose of RT in inhibiting the positive CR, and the dose of RT gradually decreases with increasing Dex use. To that end, titration of the RT dose based on the Dex dose is advised. Based on ED95, the optimal dose of RT was estimated to be 0.15 and 0.09 mg/kg when Dex was 0.5 or 1 µg/kg, respectively. Shenqiang [21] also believed that RT had a good synergistic effect with Dex since it induced and maintained a significantly lower dose for a shorter time to loss of consciousness. However, their study included sedation during bronchoscopy, and the specific dose of the drug was unknown.

Age affects the efficacy of anesthetics [22, 23], particularly for elderly patients with cardiovascular diseases such as hypertension, therefore, cardiovascular adverse events are more likely to occur during anesthesia induction [24, 25]. However, there are no studies on the effect of age on the induction of double-lumen endobronchial anesthesia with RT. As stated by our findings, elderly patients are more sensitive to RT doses, and lower RT doses can meet induction needs without leading to a positive CR. According to the ED95 estimation, the optimal drug doses for young and elderly patients using RT alone were 0.23 and 0.18 mg/kg, respectively. Chae [22] et al. also found a significant association between older age and lower ED50/ED95, with an optimal dose of 0.19 mg/kg of RT for loss of consciousness in patients aged 60–80 years. The elderly patients in our study were defined as those over the age of 65 and were administered 0.5 µg/kg Sufentanil before using RT so that the optimal dose was 0.18 mg/kg, which was slightly lower than the doses reported in the other studies. To explore the optimal sedation dose of RT in gastrointestinal endoscopy in young patients, Cao [26] et al. intravenously administered Sufentanil 0.15 µg/kg 2 min before administration of RT and found the ED95 is 0.107 mg/kg, which is recommended at a lower dose given the combined use of Sufentanil and low level of sedation.

RT has a lower effect on circulatory function than Propofol [27], only one case of hypotension was observed during this study. On the contrary, Kim [28] et al. reported 46% of cases of hypotension when exploring the optimal dose of RT for laryngeal mask placement. This result could be attributed to the use of female subjects, the whole operation time observed, and the more stringent definition of hypotension. This study found that RT could cause a decrease in MAP and HR. Older patients should have been more sensitive to RT, but the decline in the A-O group was close to that in the A-Y group. This is because the dose of RT used in the A-O is lower than that in the A-Y group, so it is suggested that rational drug use can reduce the circulatory inhibitory effect of RT in elderly patients. Dex was not used in group A-Y, and the increase in blood pressure and heart rate before and after intubation was greater than that in groups B and C. So, the magnitude of the circulatory fluctuations caused by intubation was also greater in the absence of Dex, confirming its role in reducing CR and stabilizing circulation.

Our study has certain limitations. First, our study included subjects by Dixon up-and-down method and was not randomized. Therefore, the hemodynamic-related variables between groups could not be compared without bias. Second, the use of sufentanil restricted the results of our study. Sufentanil at 0.5 µg/kg was intravenously administered before using RT in our study. Opioids have synergistic effects with intravenous anesthetics. Moreover, the ED50/ED95 will increase if the synergistic effects of opioids are omitted. Simultaneously, the use of fixed-dose sufentanil has limited the popularization of the results in various opioid applications. Finally, we did not include subjects of all ages. Thus, the results do not apply to everyone.

## Conclusion

The amount of RT varied for different age groups and medication scenarios. The optimal dose of RT to inhibit positive CR induced by double-lumen bronchial intubation in elderly patients was 0.18 mg/kg and 0.23 mg/kg in younger patients. Pre-use of Dex can reduce the amount of RT. When the pre-use dose of Dex was 0.5 µg/kg, the optimal dose to inhibit positive CR of RT was 0.15 mg/kg. And, when the pre-use dose of Dex was 1 µg/kg, the optimal dose of RT was 0.9 mg/kg.

### Electronic supplementary material

Below is the link to the electronic supplementary material.

Supplementary Material 1

## Data Availability

All of the data are included in the article. Further inquiries may be sought from the corresponding author upon reasonable request.

## Abbreviations

Dex: Dexmedetomidine
ED50: Half-effective dose
ED95: 95%-effective dose
CI: Confidence interval
RT: Remimazolam tosilate
CR: Cardiovascular response
HR: Heart rate
MAP: Mean arterial pressure
MOAA/S: Modified Observer’s Assessment of Alertness/Sedation
dMAP: Differences in mean arterial pressure
dHR: Differences in heart rate

## References

[1] Shelley B, Licker M, Slinger P (2023). Thoracic anaesthetic research: 90 years of sustained progress. Br J Anaesth.

[2] Chen C (2022). A spray-as-you-go airway topical anesthesia attenuates cardiovascular responses for double-lumen tube tracheal intubation. BMC Anesthesiol.

[3] Su P (2022). A response surface analysis of the combination of Dexmedetomidine and Sufentanil for attenuating the Haemodynamic response to endotracheal intubation. Dose Response.

[4] Liu Z (2016). Median effective concentration of remifentanil for the inhibition of laryngoscope-induced cardiovascular responses. Exp Ther Med.

[5] El-Shmaa NS, El-Baradey GF (2016). The efficacy of labetalol vs dexmedetomidine for attenuation of hemodynamic stress response to laryngoscopy and endotracheal intubation. J Clin Anesth.

[6] Kilpatrick GJ (2021). Remimazolam: Non-clinical and Clinical Profile of a New Sedative/Anesthetic Agent. Front Pharmacol.

[7] Bruder EA et al. Single induction dose of etomidate versus other induction agents for endotracheal intubation in critically ill patients. Cochrane Database Syst Rev, 2015. 1(1): p. Cd010225. 10.1002/14651858.CD010225.pub2 PMC6517008 25568981

[8] Lee A, Shirley M (2021). Remimazolam: a review in Procedural Sedation. Drugs.

[9] Stöhr T (2021). Pharmacokinetic properties of remimazolam in subjects with hepatic or renal impairment. Br J Anaesth.

[10] Lee S (2019). Dexmedetomidine: present and future directions. Korean J Anesthesiol.

[11] Stylianou M, Flournoy N (2002). Dose finding using the biased coin up-and-down design and isotonic regression. Biometrics.

[12] Görges M (2017). Sequential allocation trial design in anesthesia: an introduction to methods, modeling, and clinical applications. Paediatr Anaesth.

[13] Pace NL, Stylianou MP (2007). Advances in and limitations of up-and-down methodology: a précis of clinical use, study design, and dose estimation in anesthesia research. Anesthesiology.

[14] Dixon WJ (1991). Staircase bioassay: the up-and-down method. Neurosci Biobehav Rev.

[15] Paul M, Fisher DM (2001). Are estimates of MAC Reliable?. Anesthesiology.

[16] Barends C (2023). Development of a pharmacokinetic and pharmacodynamic model for intranasal administration of midazolam in older adults: a single-site two-period crossover study. Br J Anaesth.

[17] Oh J (2022). Determination of the 95% effective dose of remimazolam to achieve loss of consciousness during anesthesia induction in different age groups. Korean J Anesthesiol.

[18] Wu X, et al. Expert consensus on the clinical application of Dexmedetomidine in China(2018). Volume 34. LINCHUANG MAZUIXUE ZAZHI; 2018. pp. 820–3. 8.

[19] Su M (2023). Median effective dose (ED(50)) of esketamine combined with propofol for children to inhibit response of gastroscope insertion. BMC Anesthesiol.

[20] He XY et al. Dexmedetomidine for the management of awake fibreoptic intubation. Cochrane Database Syst Rev, 2014. 2014(1): p. Cd009798. 10.1002/14651858.CD009798.pub2 PMC8095023 24442817

[21] Gao S (2023). Clinical effects of remimazolam alone or in combination with dexmedetomidine in patients receiving bronchoscopy and influences on postoperative cognitive function: a randomized-controlled trial. Int J Clin Pharm.

[22] Chae D (2022). Pharmacodynamic analysis of intravenous bolus remimazolam for loss of consciousness in patients undergoing general anaesthesia: a randomised, prospective, double-blind study. Br J Anaesth.

[23] Kruijt Spanjer MR, Bakker NA, Absalom AR (2011). Pharmacology in the elderly and newer anaesthesia Drugs. Best Pract Res Clin Anaesthesiol.

[24] Beyer K (2009). Hypertension and intra-operative incidents: a multicentre study of 125,000 surgical procedures in Swiss hospitals. Anaesthesia.

[25] Lv L (2020). Effectiveness of lidocaine/prilocaine cream on cardiovascular reactions from endotracheal intubation and cough events during recovery period of older patients under general anesthesia: prospective, randomized placebo-controlled study. BMC Geriatr.

[26] Cao YH (2022). The 50% and 95% effective doses of remimazolam tosilate with adjuvant sufentanil for sedation in patients with liver Cirrhosis undergoing oesophagogastric varices screening endoscopy. J Clin Pharm Ther.

[27] Wang X (2022). Safety and efficacy of remimazolam besylate in patients undergoing colonoscopy: a multicentre, single-blind, randomized, controlled, phase III trial. Front Pharmacol.

[28] Kim J (2023). Remimazolam dose for successful insertion of a supraglottic airway device with opioids: a dose-determination study using Dixon’s up-and-down method. Can J Anaesth.

