# Supplementary material for: Effect of pre-use of Dexmedetomidine on the effective inhibitory dose of remimazolam tosilate on positive cardiovascular response in double-lumen endobronchial intubation: a clinical study
Source: BMC Anesthesiol. 2023 Nov 23;23:382. doi: 10.1186/s12871-023-02305-8 (PMC10666458; doi:10.1186/s12871-023-02305-8)
Supplement: Supplementary file 1 — Supplementary Material 1 [file 12871_2023_2305_MOESM1_ESM.docx]

**Additional File 1**

**Supplement of pretest**

Because the smaller the drug dose, the more likely the positive cardiovascular response was. We hoped to get a minimum dose with no positive response. Therefore, the pretest was explored from the large to the small dose, and the dose with three negative cardiovascular response results for the first time was selected as the starting dose for the subsequent study.

The starting dose was different for each pretest group. Because the amount of drug was inversely associated with age, older age required a smaller drug dose. There was a positive correlation between the intravenous anesthetic drugs. The higher the dose of dexmedetomidine, the smaller the required dose of Remimazolam. For different populations and scenarios of each group, the starting dose was different.

In the pretest, each dose interval was 0.025mg. Although the spacing was relatively large, it was convenient to test the target range quickly.

**Supplement Table 1.** Pretest results.

| **Sequence** | **Group A-Y (mg/kg)** | **CR (+/-)** | **Group A-O (mg/kg)** | **CR (+/-)** | **Group B (mg/kg)** | **CR (+/-)** | **Group C (mg/kg)** | **CR (+/-)** |
| --- | --- | --- | --- | --- | --- | --- | --- | --- |
| 1 | 0.3 | - | 0.25 | - | 0.2 | - | 0.15 | - |
| 2 | 0.275 | - | 0.225 | - | 0.175 | - | 0.125 | - |
| 3 | 0.25 | - | 0.2 | - | 0.15 | - | 0.1 | - |
| 4 | 0.225 | - | 0.175 | - | 0.125 | + | 0.075 | + |
| 5 | 0.2 | + | 0.15 | - | 0.15 | - | 0.1 | - |
| 6 | 0.225 | - | 0.125 | - | 0.125 | + | 0.075 | - |
| 7 | 0.2 | + | 0.1 | + | 0.15 | - | 0.05 | + |
| 8 | 0.225 | + | 0.125 | + |  |  | 0.075 | + |
| 9 | 0.25 | - | 0.15 | - |  |  | 0.1 | - |
| 10 | 0.225 | + | 0.125 | + |  |  |  |  |
| 11 | 0.25 | - | 0.15 | - |  |  |  |  |

CR: Cardiovascular Response; + : Positive Cardiovascular Response; - : Negative Cardiovascular Response.
